# Etranacogene dezaparvovec in people with hemophilia B with preexisting adeno-associated virus 5 neutralizing antibodies: 4-year subgroup results from the HOPE-B trial

**DOI:** 10.1016/j.rpth.2026.103360

**Published:** 2026-01-19

**Authors:** Robert Klamroth, Paul E. Monahan, Paul Van der Valk, Doris Quon, Rashid Saeed Kazmi, Michiel Coppens, Niamh O’Connell, Steven W. Pipe, Annette von Drygalski, Saira Afzal, Richard Gabriel, Loubna Youssar, Sean Gill, Nathalie Jansen, Fei Wang, Sandra Le Quellec, Cedric Hermans

**Affiliations:** 1 Internal Medicine, Vascular Medicine and Coagulation Disorders, Vivantes Clinic Friedrichshain, Berlin, Germany; 2 CSL Behring, King of Prussia, Pennsylvania, USA; 3 Centre for Benign Haematology, Thrombosis and Haemostasis, Van Creveldkliniek, Pathology, University Medical Center Utrecht, Utrecht University, Utrecht, The Netherlands; 4 Orthopedic Hemophilia Treatment Center, The Luskin Orthopedic Institute for Children, Los Angeles, California, USA; 5 Department of Hematology, University Hospital Southampton National Health Service Foundation Trust, Southampton, United Kingdom; 6 Department of Vascular Medicine, Amsterdam University Medical Centers, University of Amsterdam, Amsterdam, The Netherlands; 7 Amsterdam Cardiovascular Sciences, Pulmonary Hypertension & Thrombosis, Amsterdam, The Netherlands; 8 National Coagulation Centre, Department of Hematology, St. James's Hospital, Dublin, Ireland; 9 School of Medicine, Trinity College, Dublin, Ireland; 10 Departments of Pediatrics and Pathology, University of Michigan, Ann Arbor, Michigan, USA; 11 Division of Hematology/Oncology, Department of Medicine, University of California San Diego, San Diego, California, USA; 12 ProtaGene CGT GmbH, Heidelberg, Germany; 13 Department of Internal Medicine, Cliniques universitaires Saint-Luc, Brussels, Belgium

**Keywords:** hemophilia B, antibodies, neutralizing, factor IX, humans, genetic therapy

## Abstract

**Background:**

Health Outcomes with Padua Gene; Evaluation in Hemophilia B (HOPE-B) is the first phase 3 trial of adeno-associated virus (AAV) serotype 5 vector-based gene therapy for hemophilia B to have enrolled participants with neutralizing antibodies (NAbs) to the viral vector.

**Objectives:**

Evaluate the efficacy and safety of etranacogene dezaparvovec in a post hoc subgroup of participants with preexisting AAV5 NAbs 4 years posttherapy.

**Methods:**

After a ≥6-month lead-in period on continuous prophylaxis, people with moderately severe/severe hemophilia B (factor [F]IX ≤ 2%) received a single infusion of etranacogene dezaparvovec. AAV5 NAb status prior to infusion was determined. FIX activity, annualized bleeding rates (ABRs), and safety were evaluated in participants with preexisting AAV5 NAbs.

**Results:**

A total of 21/54 participants had detectable preexisting AAV5 NAbs (titer: 8.5-678, *n* = 20; titer: 3212, *n* = 1). Two participants did not respond to treatment (high titer: 3212, *n* = 1; received partial dose, *n* = 1). The mean FIX activity was 36 IU/dL (SD, 18) at 1 year (*n* = 18) and 34 IU/dL (SD, 16) at 4 years (*n* = 15) posttreatment. Unadjusted ABRs for all bleeds (treated and untreated) decreased by 74.4% from 4.64 during lead-in to 1.18 during months 7 to 48 (*n* = 21). ABRs for spontaneous and joint bleeds decreased by 79% and 82%, respectively. The mean FIX consumption decreased from 245,476 IU/y (SD, 144,497) during lead-in to 23,975 IU/y (SD, 48.928) during years 1 to 4 (*P* < .0001). The most frequent treatment-related adverse events were infusion-related reactions (23.8%) and transient alanine aminotransferase elevations (14.3%). No late hepatotoxicity was observed.

**Conclusion:**

Etranacogene dezaparvovec demonstrated long-term efficacy and safety in individuals with preexisting AAV5 NAb titers ≤ 678, expanding eligibility for gene therapy to a broader hemophilia B population.

## Essentials

• Conventionally, people with antibodies to viral vectors were excluded from gene therapy trials.
• Patients with antibodies to the vector were included in HOPE-B and treated with etranacogene dezaparvovec.
• The 4-year follow-up showed sustained FIX activity and lasting reduction in bleeding rates.
• Etranacogene dezaparvovec is safe, with no late hepatotoxicity detected post-therapy.

## Graphical abstract

**Figure:**
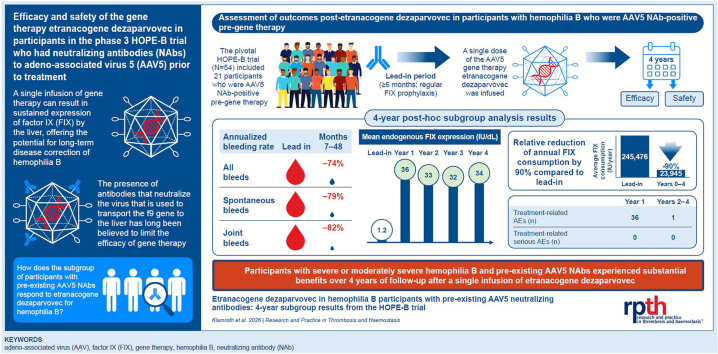

## 1 Introduction

Gene therapy has the potential to revolutionize the treatment of hemophilia B, a rare bleeding disorder that is characterized by a congenital deficiency of clotting factor (F)IX [1,2]. Although evidence indicates that adeno-associated virus (AAV) vector-based gene therapy results in sustained correction of FIX activity in individuals with hemophilia B [[3], [4], [5]], concerns have been raised regarding the possibility that humoral immunity to the viral vectors used could affect delivery of the therapeutic gene to target tissues [[5], [6], [7], [8], [9], [10]].

Etranacogene dezaparvovec (CSL222, HEMGENIX, CSL Behring) is an AAV5 vector-based gene therapy containing the FIX-Padua gene expression cassette. Its serotype-derived liver tropism and hepatocyte-specific promoter direct FIX expression to the liver, which is the natural site of FIX production [4]. Individuals with detectable AAV5-neutralizing antibodies (NAbs) were initially excluded from participation in the AMT060 phase 1/2 trial of AAV5-based gene therapy that drives the expression of wild-type human FIX for hemophilia B [11]. A switch from a green fluorescent protein- to a luciferase-based NAb assay revealed, in hindsight, that 3 of the 10 participants had AAV5 NAbs at the time of dosing. However, these participants expressed FIX with no correlation between NAb titer and endogenous FIX activity [11]. Based on these results, the initial phase 2b trial employing the FIX-Padua gene variant was conducted in 3 participants, irrespective of their AAV5 NAb status. All had detectable AAV5 NAbs at baseline and sustained endogenous FIX-Padua production in the high-mild/non-hemophilia range for 5 years, so that positivity was no longer considered an exclusion criterion for trials of etranacogene dezaparvovec [5]. The pivotal phase 3 HOPE-B trial (NCT03569891, CSL Behring) investigated the efficacy and safety of etranacogene dezaparvovec in individuals with severe or moderately severe hemophilia B, regardless of preexisting NAbs. Participants expressed sustained endogenous FIX, with mean FIX activity levels of 41.5 IU/dL (SD, 21.7) and 36.7 IU/dL (SD, 19.0) at 12 and 24 months, respectively. The sustained endogenous FIX activity translated into reduced adjusted annualized bleeding rates (ABRs) for all bleeds (treated and untreated) of 1.51 between 7 and 24 months post-treatment, compared with 4.18 during lead-in while on standard-of-care FIX prophylaxis, and reduced exogenous FIX consumption over 24 months after a single etranacogene dezaparvovec infusion [8,12]. Health Outcomes with Padua Gene; Evaluation in Hemophilia B (HOPE-B) also demonstrated that etranacogene dezaparvovec has a favorable safety profile [8,12]. The HOPE-B trial is the only phase 3 gene therapy trial for hemophilia that included participants with preexisting AAV5 NAbs (NAb+) [8,12]. This offers a rare and clinically meaningful opportunity to evaluate the efficacy and safety of etranacogene dezaparvovec, including the interindividual variability of these endpoints, in a population historically excluded from such interventions, providing critical insights to inform treatment decisions and clinical practice, which is the aim of this post hoc analysis.

## 2 Methods

### 2.1 Study participants

A detailed description of the HOPE-B inclusion and exclusion criteria and study design has been published previously [12]. In brief, participants were adult males (≥18 years) with either severe (plasma FIX activity < 1 IU/dL) or moderately severe (plasma FIX activity 1-2 IU/dL) hemophilia B. Eligible participants were on stable, continuous exogenous FIX prophylaxis, with the specific dose and product determined by their physician. Key exclusion criteria were a history of FIX inhibitors, active hepatitis B or C infection, other known severe infections, liver fibrosis detected by elastography, and/or another significant uncontrolled concurrent medical condition. Prior to treatment with etranacogene dezaparvovec, the AAV5 NAb status of all enrolled participants was established but not used as an exclusion criterion. This post hoc analysis includes only participants enrolled in HOPE-B who were NAb+ on the day of the infusion. Neither patients nor investigators knew the patient’s NAb status and NAb titer prior to etranacogene dezaparvovec infusion.

### 2.2 Study design and endpoints

HOPE-B was a phase 3, open-label, multinational study conducted at 33 sites. After a lead-in period of ≥6 months during which participants remained on their usual continuous exogenous FIX prophylaxis, eligible participants received a single intravenous infusion of etranacogene dezaparvovec (2 × 10^13^ gc/kg body weight).

The primary endpoint of HOPE-B was the ABR during a 52-week period from months 7 to 18 post-treatment. Secondary endpoints included FIX activity levels (measured by a one-stage activated partial thromboplastin time-based assay [SynthasIL, Instrumentation Laboratory Company]) during the 52 weeks following establishment of stable FIX expression (months 7-18) post-treatment, the use of exogenous FIX replacement, the frequency and severity of adverse events (AEs), and the reactive use of corticosteroids for management of transaminitis. Patient follow-up for 5 years post-treatment is planned. Here, we report analyses of ABRs, FIX activity, exogenous FIX use, and safety over 4 years of follow-up after administration of etranacogene dezaparvovec to NAb+ participants at baseline.

### 2.3 Analysis of AAV5 NAbs

Serum samples for AAV5 NAb determination were obtained from participants during screening, lead-in (at 8 and 4 weeks prior to etranacogene dezaparvovec administration), and on the day of etranacogene dezaparvovec administration (ie, baseline NAb status). The AAV5 NAb assay measured the potential of the participant’s serum to inhibit the transduction of mammalian cells by the AAV5 vector expressing luciferase *in vitro*. A cell-based NAb assay with a starting serum dilution of 1:2 and 7 dilution steps was used for both screening and measuring NAb titers, as described previously [8,12,13]. The lower limit of detection was a NAb titer of 7. Central laboratories assessed AAV5 immunoglobulin (Ig) G antibodies (Unilabs) and AAV5 NAbs (Precision for Medicine). Total IgG antibodies directed against AAV5, whether or not they had neutralizing activity, were measured using an enzyme-linked immunosorbent assay, as described previously [13]. Up to 3 separate assays for IgG were used for screening, confirmation, and measuring titers (titering), respectively. The IgG titering assays had a starting serum dilution of 1:50 and 8 dilution steps.

### 2.4 Molecular analyses to assess oncogenicity and genotoxicity

Molecular analyses to detect vector integration sites (IS), were conducted by ProtaGene CGT GmbH independently of the sponsor, using DNA samples extracted from tumor tissue and blood. A detailed description of the analyses can be found in the Supplementary Methods.

Briefly, DNA was extracted using the QIAamp DNA Mini Kit (Qiagen). A polymerase chain reaction with vector-specific primers (hFIXco_FW and hFIXco_RV) was performed on 10 ng of DNA per sample, with vector-containing plasmids as a positive control. Whole-genome sequencing data were analyzed to detect IS and perform somatic variant calling. In addition, target enrichment sequencing was used to selectively enrich for AAV vector sequences and determine vector IS.

### 2.5 Statistical analysis

Demographic and baseline characteristics were summarized descriptively using sample size (*n*), mean, SD, minimum, maximum, median, and IQR for continuous measurements, and frequency and percentages for categorical variables.

Both unadjusted and adjusted ABRs were calculated. Unadjusted ABRs were calculated as the sum of the number of bleeds divided by the sum of person-time at risk (in years) during a given time period across all participants. Adjusted ABRs were estimated for the lead-in and post-treatment periods using generalized estimating equations, assuming bleeding events follow a negative binomial distribution and an offset parameter equal to the natural log of the collection period (years). An unstructured covariance matrix was specified, and treatment was included as a categorical variable. If convergence failed, then a compound symmetry covariance structure was used. If convergence was not attained, then initial parameter estimates were provided. The estimated rate ratio, one-sided 97.5% Wald CI, and corresponding *P* value were determined. Any person-time during the post-treatment period, within 5 half-lives after exogenous FIX use, was not counted as time at risk of having a bleeding event. However, any unique bleeding events on or after stable FIX expression were counted as events, even if they occurred during a period of exogenous FIX contamination. All bleeding events during the lead-in period were counted, and the entire lead-in period was assumed to be time at risk. Additionally, an analysis of adjusted ABRs, including only participants who expressed endogenous FIX pos-tgene therapy (responder analysis set), was performed.

Descriptive statistics were used to report one-stage activated partial thromboplastin time-based (SynthasIL) FIX activity measurements (expressed as IU/dL) from the central laboratory. Post-gene therapy FIX samples were considered contaminated with exogenous FIX and excluded from analysis if drawn within 5 half-lives of FIX concentrate administration, based on the reported half-life of each product.

A potential correlation between individual AAV5 NAb titers at baseline and FIX expression 7 to 48 months post-etranacogene dezaparvovec infusion was analyzed using a simple linear regression with FIX activity as the outcome and the base-10 logarithm of the AAV5 titer at baseline as the lone covariate.

Annualized FIX consumption, excluding FIX replacement for invasive procedures, was computed for each period by dividing the total consumption by the time under observation (in years), and compared between the treatment and lead-in phase using a 2-sided paired *t*-test (using the pair of values from each participant).

The analysis describes a retrospective examination of data from the HOPE-B trial collected during the lead-in period, after all patients included in this analysis had reached 4 years of follow-up, and were not specifically powered to detect significant differences or associations. All analyses were performed in SAS 9.4 (SAS Institute).

## 3 Results

### 3.1 Study participants

**Figure 1:**
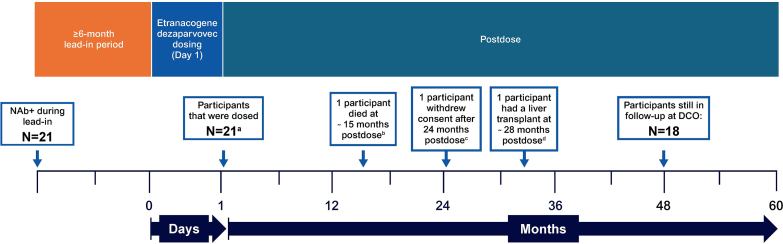
Disposition of participants. ^a^One participant discontinued the infusion of etranacogene dezaparvovec due to a treatment-emergent adverse event of hypersensitivity after receiving ∼10% of the full dose. This participant was included in the full analysis set and the safety analyses but excluded from the responder analysis set. ^b^This participant had a non–treatment-related treatment-emergent adverse event of cardiogenic shock that resulted in death at 464 days postdose. Efficacy and safety data from this participant before the event were included in the analyses for the full analysis set, responder analysis set, and safety population set. ^c^This participant had a baseline adeno-associated viral vector serotype 5 titer of 3212 and did not respond to treatment with etranacogene dezaparvovec. Before the participant withdrew consent, his efficacy and safety data were included in the analyses for the full analysis set and the safety population set but were excluded from the responder analysis set. He continues to be followed for safety through review of medical records. ^d^Before the liver transplant, efficacy and safety data from this participant were included in the analyses for the full analysis set, responder analysis set, and safety population set. After the liver transplant, hepatocytes from the transplanted liver produced endogenous wild-type factor IX. As such, efficacy data from this participant no longer informed outcomes from gene therapy and were excluded from the analysis. Safety data from this participant were included in the analyses regardless of the liver transplant. DCO, data cutoff; NAb+, with preexisting adeno-associated viral vector serotype 5 neutralizing antibodies.

Of the 54 participants included in the HOPE-B trial, 21 had detectable AAV5 NAbs on the day of dosing, and 20 of these received the full dose of etranacogene dezaparvovec. As described previously [8,12], 1 participant (baseline FIX < 1 IU/dL and AAV5 NAb titer: 198.9) prematurely discontinued etranacogene dezaparvovec infusion following a treatment-emergent AE of hypersensitivity, having received approximately 10% of a full dose. Following gene therapy, his first uncontaminated one-stage FIX activity level was 1.7 IU/dL at the central laboratory and 1 IU/dL at the local laboratory at week 11 (41 days after prior exogenous FIX infusion). As a result, the participant did not discontinue regular FIX prophylaxis infusions and was considered not to have responded to etranacogene dezaparvovec treatment, but continued to participate in the trial [12]. One participant died at day 464 postdose due to an AE not related to study treatment [8]. Two additional participants were excluded from the efficacy analyses at 48 months: 1 due to a liver transplant and 1 due to withdrawal of consent for efficacy assessments (the patient with the highest AAV5 NAb titer of 3212; Figure 1). Therefore, all 21 participants were included in efficacy analyses through 24 months, 20 in analyses for 24 to 36 months, and 18 in analyses for 36 to 48 months post-gene therapy. All 21 participants were included in the safety analysis set, but the participant who died at day 464 was excluded from the safety analysis after 18 months post-gene therapy. The baseline demographic and clinical characteristics of NAb+ participants are summarized in Table 1.

**Table 1:** Participant baseline demographics and clinical characteristics.

Characteristic	AAV5 NAb+ participants (*n* = 21), *n* (%)
Age at screening (y), mean (SD; min-max)	44.5 (17.5; 19-75)
Positive HIV status	1 (4.8)
Prior hepatitis B	5 (23.8)
Prior hepatitis C	14 (66.7)
Severity of hemophilia B at diagnosis	
Severe (FIX < 1%)	16 (76.2)
Moderately severe (FIX ≥ 1% and ≤2%)	5 (23.8)
Prescreening for FIX treatment	
Extended half-life	14 (66.7)
Standard half-life	7 (33.3)
AAV5 NAb titer on the day of infusion	
Maximum	3212
Median (IQR)	57 (23-199)
Participants with zero reported bleeds during the lead-in period	3 (14.3)

AAV5, adeno-associated viral vector serotype 5; FIX, factor IX; NAb, neutralizing antibody.

### 3.2 AAV5 NAbs and total antibodies to AAV5

The median AAV5 NAb titer on the day of dosing prior to infusion was 57 (IQR, 23-199); 20/21 participants had detectable AAV5 NAbs, ranging from a titer of 8.5 to 678, and 1 participant had a titer of 3212 (Table 1). All but 2 participants had detectable NAbs at screening and throughout the lead-in period; the 2 participants in whom NAb levels were below the limit of detection at screening developed NAbs by month 4 during the lead-in period (NAb titer: 82.5) and by the day of dosing prior to infusion (NAb titer: 13.7), respectively [13].

Of the 21 NAb+ participants, 14 had detectable IgG antibodies on the day of dosing prior to infusion. IgG titers ranged from <50 to 8081. Detectable AAV5 NAb and IgG titers were correlated at all time points during lead-in [13].

Post-gene therapy, NAb titers exceeded the upper limit of quantification (8748) in all participants within 3 weeks of dosing and remained above this limit throughout the 4 years of follow-up. Similarly, IgG titers were increased 3 weeks after dosing and remained high throughout the 4 years of follow-up, exceeding the upper limit of quantification in most participants.

### 3.3 Endogenous FIX activity

Participants in HOPE-B, including NAb-negative (NAb−) participants (*N* = 54), expressed sustained endogenous FIX at 48 months, with a mean FIX activity level of 37 IU/dL (SD, 17; data on file).

Of the NAb+ population, 2 participants with baseline FIX < 1 IU/dL at the time of diagnosis demonstrated minimal evidence of hepatic transduction with the FIX-Padua transgene. Uncontaminated endogenous FIX activity for the participant with the highest NAb titer was 1.5 IU/dL at the central laboratory (1 IU/dL at the local laboratory) at week 5; uncontaminated endogenous FIX activity for the participant who received ∼10% of the etranacogene dezaparvovec dose was 1.7 IU/dL at the central laboratory (1 IU/dL at the local laboratory) at week 11. These individuals were considered nonresponders because their uncontaminated FIX levels were ≤2.0 IU/dL (ie, inclusion criteria) and they did not discontinue regular FIX prophylactic infusions post-treatment with etranacogene dezaparvovec as per protocol guidance. The participant who had a liver transplant 28 months post-treatment did not express etranacogene dezaparvovec transgene-derived FIX after receiving the transplant. His last uncontaminated endogenous transgene-derived FIX activity prior to the liver transplant was 37 IU/dL, and his baseline NAb titer was 14.9. Finally, 1 patient returned to routine FIX prophylaxis between months 29 and 30 post-gene therapy. His baseline NAb titer was 98.5.

**Figure 2:**
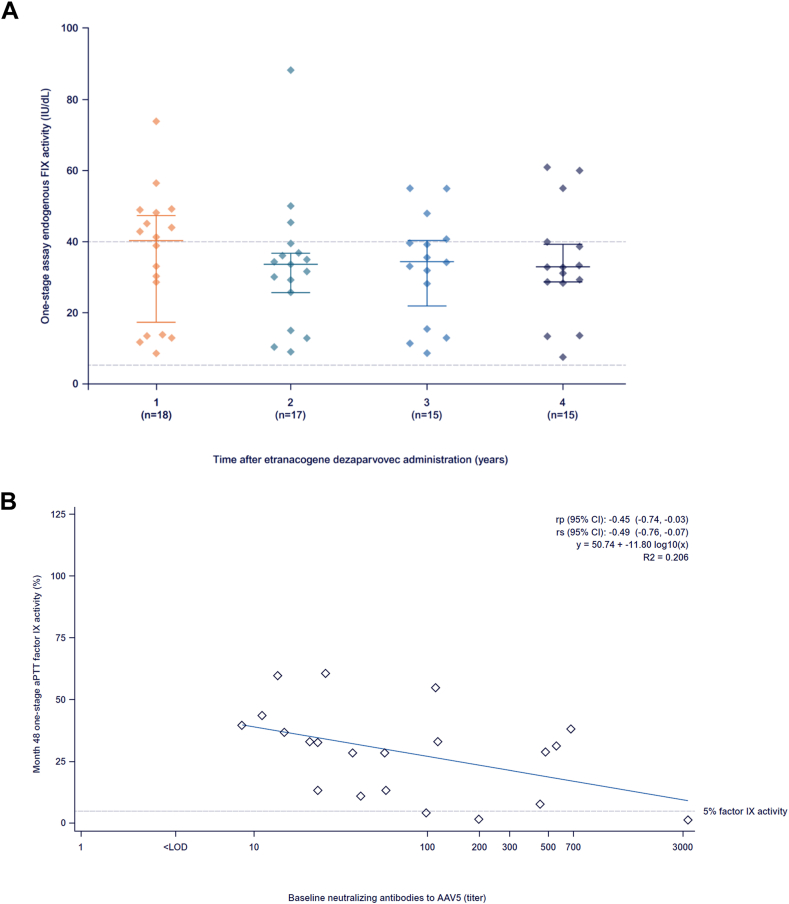
Endogenous factor (F)IX activity (A) over the 4-year follow-up and (B) as a function of baseline adeno-associated viral vector serotype 5 (AAV5) neutralizing antibody titer. The latter was calculated for the entire posttreatment period (months 7-48). Horizontal lines within the boxes in A depict medians, bars depict upper and lower quartiles (quartiles 1-3), and diamonds show individual levels of expression at years 1 to 4. aPTT, activated partial thromboplastin time; rp, Pearson product-moment correlation coefficient; rs, Spearman correlation coefficient.

The mean endogenous FIX activity was 36 IU/dL (SD, 18) at year 1 (*n* = 18; no sample: *n* = 1; sample contaminated due to exogenous FIX use: *n* = 2) and 34 IU/dL (SD, 16) at year 4 (*n* = 15; no sample: *n* = 4, including the participant who underwent a liver transplant; sample contaminated due to exogenous FIX use: *n* = 2) post-etranacogene dezaparvovec infusion (Figure 2A). The median endogenous FIX activity at year 4 was 33 IU/dL (range, 8-61; Figure 2A and Supplementary Figure).

Notably, no clinically meaningful correlation between individual AAV5 titer at baseline and the median endogenous FIX activity from months 7 to 48 was identified (Figure 2B).

### 3.4 ABR

**Figure 3:**
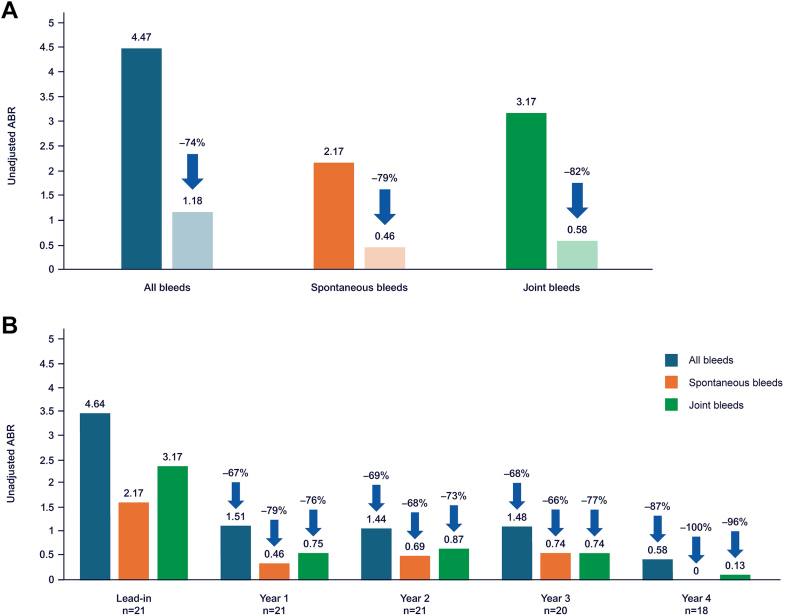
Unadjusted annualized bleeding rates (ABRs) for (A) all bleeds (treated and untreated), spontaneous bleeds, and joint bleeds during the lead-in period vs months 7 to 48 (*n* = 21), and (B) during the lead-in period vs years 1 to 4.

The unadjusted ABR for all bleeds (treated and untreated) decreased by 74.4% from 4.64 during lead-in to 1.18 during months 7 to 48 post-treatment (*n* = 21). The unadjusted ABR for spontaneous bleeds decreased from 2.17 during lead-in to 0.46 during months 7 to 48 post-treatment with etranacogene dezaparvovec, with all participants free of spontaneous bleeds in year 4 (Figure 3). For joint bleeds, the unadjusted ABR decreased from 3.17 during lead-in to 0.58 during months 7 to 48 post-treatment. Unadjusted ABRs for all, spontaneous, and joint bleeds for each year of follow-up are shown in Figure 3.

Regarding adjusted ABRs, the participant who received ∼10% of the planned dose and the one with the highest AAV5 NAb titer experienced a total of 6 and 5 bleeding episodes, respectively, during months 7 to 48, during which time each was receiving regular continuous FIX prophylaxis. The trial’s prespecified statistical rules generated aberrantly elevated adjusted ABRs for these 2 nonresponder participants (eg, a clinically anomalous calculated individual ABR of >1600), obscuring the ability to interpret adjusted ABR results for the gene therapy cohort, as explained in the Supplementary Results and shown in Supplementary Tables S1 and S2. The adjusted ABR for the responder analysis set was significantly reduced during months 7 to 48 post-etranacogene dezaparvovec treatment compared with the lead-in period (0.52 [95% CI, 0.23, 1.19] vs 3.05 [95% CI, 2.03, 4.56]; *P* < .0001).

### 3.5 Use of exogenous FIX post-gene therapy

**Figure 4:**
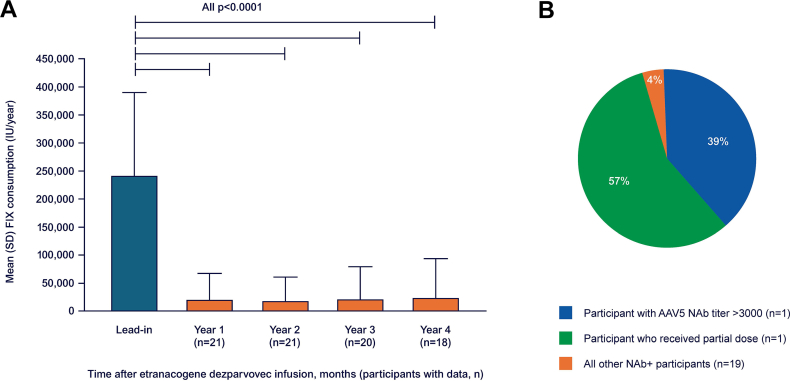
(A) Mean exogenous factor (F)IX consumption (IU/y) during the lead-in period compared with years 1 to 4 postetranacogene dezaparvovec treatment, and (B) relative contribution of participants to mean FIX consumption (IU/y) over 4 years posttreatment. AAV5, adeno-associated viral vector serotype 5; NAb, neutralizing antibody; NAb+, with preexisting AAV5 NAbs at baseline.

FIX consumption significantly decreased by 90%, from a mean of 245,476 IU/y (SD, 144,497) during lead-in to 23,975 IU/y (SD, 48,928) during years 0 to 4 post-gene therapy (*P* < .0001) and remained stable over time (Figure 4A). Most of the post-gene therapy exogenous FIX use was due to the 2 participants who remained on FIX prophylaxis, who received 162,600 IU/y and 109,830 IU/y over 4 years post-treatment, respectively (Figure 4B). The one participant who returned to prophylaxis in year 3 received 199,590 IU/y of exogenous FIX product in year 3 and 256,175 IU/y in year 4. No participant returned to prophylaxis in year 4, and 13 of 18 (72%) participants who remained free from FIX prophylaxis did not receive any exogenous FIX infusions. A total of 8 participants did not receive any exogenous FIX infusions throughout the entire post-treatment period.

### 3.6 Safety

All 21 NAb+ participants who received etranacogene dezaparvovec experienced AEs during the 48 months post-treatment. Of the 308 treatment-emergent AEs reported by NAb+ participants, 68.5% were mild, 17.9% were moderate, and 6.8% were severe. Treatment-related AEs occurred in 71.4% of NAb+ participants (Table 2). Most of these events occurred within the first 3 months post-treatment. The 1 participant who returned to prophylaxis during year 3 did so due to a treatment-related AE, of specific *f9* gene expression between months 29 and 30 post-gene therapy. No other treatment-related AEs, no inhibitor development, or thrombotic events were reported during months 3 to 48 of follow-up.

**Table 2:** Treatment-related adverse events in the posttreatment period experienced by ≥5% of NAb+participants.

AE by preferred term	Participants with an AE (*n* = 21), *n* (%)	No. of events
Participants with at least 1 treatment-related AE	15 (71.4)	36
Influenza-like illness	4 (19.0)	4
ALT increased	3 (14.3)	3
IRR	5 (23.8)	5
ALP increased	2 (9.5)	2
CRP increased	2 (9.5)	2
Headache	2 (9.5)	2
Nausea	2 (9.5)	2

AE, adverse event; ALP, alkaline phosphatase; ALT, alanine aminotransferase; CRP, C-reactive protein; IRR, infusion-related reaction; NAb+, neutralizing antibody-positive.

### 3.7 Infusion-related reactions

The incidence of infusion-related reactions (IRRs) in NAb+ participants was 5/21 (23.8%), with 4 having AAV5 NAb titers ≥ 198 and 1 having an AAV5 NAb titer of 23. One of these participants (AAV5 NAb titer: 198.9), the second participant infused in the trial, experienced an IRR of moderate severity; after having received supportive care for a suspected hypersensitivity reaction, gene therapy administration was withdrawn after an estimated 10% of the dose had been infused. This participant did not subsequently express FIX (ie, he experienced a lack of efficacy of gene transfer) and did not discontinue continuous FIX prophylaxis. All other AEs of IRRs were reported as mild (*n* = 4) or moderate (*n* = 1); management involved either observation only or pausing dosing, followed by reinitiation at a slower infusion rate, with or without symptomatic support using an antihistamine and single-dose hydrocortisone.

### 3.8 Liver transaminase elevation

The most frequently observed AE after IRRs was transient alanine aminotransferase (ALT) elevation (*n* = 3; 14.3%), all within the first 3 months post-gene therapy (Supplementary Figure). These ALT elevations were first observed on study days 24, 28, and 35, respectively. Two participants had an increase in ALT levels 1- to 2-fold the upper limit of normal, and ALT levels increased to >2-fold the baseline levels but remained within the normal range in 1 participant. These 3 participants received a reactive course of corticosteroid treatment, starting 0 to 10 days following ALT elevation. The median maximum duration of ALT elevation was 17 days (range, 15-42), and corticosteroids were used for a median of 83 days (range, 56-117), including tapering. Two participants had endogenous FIX activity levels of 16 IU/dL and 5 IU/dL at or near the time of corticosteroid initiation, respectively, and 13 IU/dL at year 4 and 11 IU/dL at month 42 (the value at year 4 was contaminated by episodic FIX exogenous use prior to the study visit), respectively. The remaining participant had endogenous FIX levels of 16 IU/dL at or near the time of corticosteroid initiation, 9.1 IU/dL at the end of the corticosteroid tapering (∼month 3 post-gene therapy), 13 IU/dL at month 24, and 4 IU/dL at month 30 without recurrence of ALT elevation before resuming continuous FIX prophylaxis.

No signs of late or long-term hepatotoxicity were observed.

### 3.9 Malignancy and genotoxicity

Two serious AEs unrelated to treatment have been reported previously (hepatocellular carcinoma, *n* = 1; death, *n* = 1) [8,12,14]. During year 4, 1 serious AE (myelodysplastic syndrome [MDS], *n* = 1) was observed in 1 NAb+ participant and explored by molecular analysis for vector integration. No evidence of AAV5 vector DNA in tumor or blood samples from this participant was detected by polymerase chain reaction using vector-specific primers; no integration events were identified in affected tissues by whole-genome or target enrichment sequencing, while a premalignant genetic signature consistent with the development of this disease was found. The MDS was considered unrelated to treatment. A detailed description of the patient narrative, molecular analyses for IS detection, and the identification of relevant genetic signatures and corresponding findings are provided in the Supplementary Text. No treatment-related oncogenicity was observed up to 4 years post-treatment.

## 4 Discussion

The HOPE-B trial represents a pivotal advancement in the field of gene therapy for hemophilia B, challenging a longstanding paradigm that pre-existing AAV NAbs pose an insurmountable barrier to the successful systemic delivery of *in vivo* gene therapy. Historically, individuals with detectable AAV NAbs have been systematically excluded from AAV-based clinical trials, based on the premise that humoral immunity would compromise vector transduction and, consequently, therapeutic efficacy [9,15]. This exclusion reflected concerns that NAbs could intercept AAV particles, thereby diminishing their ability to deliver genetic payloads to target cells. HOPE-B is the first phase 3 trial to enroll adults with hemophilia B irrespective of their AAV5 NAb status. The post hoc analysis provided in the present study specifically evaluated efficacy and safety outcomes among participants with detectable NAbs prior to administration of etranacogene dezaparvovec. While earlier HOPE-B trial reports provide data for the entire study cohort, this analysis allows the clinical researcher to examine composite and individual outcomes for this unique study subgroup.

Etranacogene dezaparvovec demonstrated transgene expression in individuals with pre-existing NAb titers up to 678 (using the specific AAV5 NAb assay from HOPE-B) who received the full dose, indicating that this level of pre-existing AAV5 NAbs did not preclude therapeutic benefit.

The NAb+ participant with the highest recorded NAb titer (3212, as measured by the trial-specific luciferase-based assay) did not respond to gene therapy and remained on continuous FIX prophylaxis. This finding suggests that pre-existing AAV5 NAbs at this level, while likely present at a prevalence of only approximately 1% to 4% [13,[16], [17], [18]], may markedly impair vector transduction. However, data remain unavailable for individuals with AAV5 NAb titers between 678 and 3212, leaving an unresolved question regarding a potential threshold at which AAV5 NAbs become clinically consequential, ie, the range of NAb titers at which the efficacy of etranacogene dezaparvovec is uncertain in the context of this specific AAV5-mediated gene delivery. An ongoing trial recruiting people with hemophilia B and detectable AAV5 NAbs at baseline (NCT06003387) may provide further insight into this issue [19].

Except for 1 participant who experienced an unexplained late decline in transgene-derived endogenous FIX activity to 3.6 IU/dL and who eventually resumed continuous FIX prophylaxis at approximately 29 months post-treatment, all NAb+ individuals who successfully expressed the transgene-derived FIX-Padua protein maintained stable, durable FIX activity over 4 years following gene therapy and remained free from prophylaxis. This suggests that although pre-existing AAV5 NAbs may affect initial vector transduction and transgene expression, they do not impair long-term FIX protein durability after successful transduction, even though AAV5 NAb levels remain high post-gene therapy.

A central question emerging from these observations concerns the apparent serotype-specific permissiveness of AAV5-based gene therapy in the presence of pre-existing NAbs, which is in contrast to the more pronounced inhibitory effects of NAbs observed for gene therapy platforms utilizing other AAV serotypes. The literature consistently reports that, across diverse study populations and despite the use of various assay methodologies, AAV5 NAb titers are generally among the lowest observed compared with those of NAbs directed against other AAV serotypes [13,[16], [17], [18]]. This may reflect limited cross-reactivity due to the low capsid homology between AAV5 and other AAV serotypes [20]; in addition, cross-reactive NAb titers are generally lower than those against the primary infecting serotype, and individuals with low-titer NAbs against one serotype rarely neutralize other serotypes [21]. Furthermore, a study found that the binding affinity of AAV5-directed NAbs for their cognate capsid appears to be lower than that of NAbs targeting other AAV serotypes [22]. This difference in antibody-capsid interaction strength could mitigate the neutralizing effect, allowing a meaningful proportion of AAV5 vectors to evade immune sequestration and reach target cells. AAV vectors enter target cells via membrane receptors, and it is possible that these receptors bind AAV5 capsids more strongly than circulating NAbs [23], allowing vector transduction despite detectable antibody levels. This mechanistic distinction may explain why AAV5 vectors can achieve efficient gene delivery in NAb+ individuals, whereas vectors based on other serotypes are more predictably neutralized by pre-existing antibodies. Other explanations, such as bypassing neutralization with a high vector dose, have been suggested [24]. However, the absence of a clinically meaningful correlation between AAV5 NAb titer (up to a threshold of 678, as observed in this trial) and FIX activity refutes the hypothesis that the efficacy of etranacogene dezaparvovec in NAb+ individuals is solely dose-dependent.

These findings are further contextualized by observations from another clinical trial evaluating an AAV5 gene therapy for the treatment of hemophilia A (ie, FVIII deficiency), valoctocogene roxaparvovec. In the pivotal trials of valoctocogene roxaparvovec, eligibility criteria excluded individuals with preexisting total antibodies against AAV5 rather than specifically NAbs. Notably, participants in the treated population who harbored detectable AAV5 NAbs at baseline demonstrated meaningful increases in FVIII activity following vector administration [25]. This reinforces the assumption that the permissiveness of AAV5 vectors in the setting of preexisting NAbs is not unique to the HOPE-B trial or to etranacogene dezaparvovec, but instead reflects a broader serotype-specific phenomenon.

Demonstrating that etranacogene dezaparvovec, an AAV5-based vector, retains efficacy in individualswth pre-existing AAV5 NAbs is particularly important, as it enables more patients to benefit from this gene therapy.

Following etranacogene dezaparvovec administration, the ABRs for all bleeds among NAb+ participants significantly decreased compared with the lead-in period, during which participants received standard continuous FIX prophylaxis. Among those who discontinued FIX prophylaxis after gene therapy, the adjusted ABR (all treated and untreated bleeds) showed a significant reduction from 4.43 during the lead-in period to 1.13 during months 7 to 48 post-treatment (Supplementary Table S1). This improvement was consistent across all bleed categories, including total, spontaneous, and joint bleeds. Importantly, these efficacy outcomes in NAb+ participants mirrored those observed in the overall HOPE-B trial population, including both NAb+ and NAb− individuals, in which ABR for all bleeds decreased from 4.16 during the lead-in period to 1.63 during months 7 to 48 post-treatment [3].

Etranacogene dezaparvovec has consistently demonstrated a favorable safety profile over 4 years post-treatment across the clinical development program, including the HOPE-B trial cohort of NAb+ participants.

Although a higher likelihood of IRRs among participants with baseline NAbs cannot be excluded, given that 5 of the 7 IRRs reported in the HOPE-B trial occurred in NAb+ participants [8,12], particularly those with higher NAb titers, current data indicate that these events were generally transient and manageable. The underlying mechanism for this potential association remains unclear but is likely multifactorial, involving processes such as complement activation triggered by immune complexes formed between AAV capsids and antibodies, as well as amplification of innate immune responses, ultimately leading to cytokine release and systemic inflammation [26,27].

Importantly, there was a low incidence or severity of ALT elevations among NAb+ participants (14%), similar to NAb− participants (18%) [28], and all within 3 months post-gene therapy, indicating that pre-existing humoral immunity does not confer an additional risk for acute or subacute hepatocellular injury following etranacogene dezaparvovec infusion. Moreover, no persistent or late-emerging hepatotoxicity was identified, supporting the hepatic safety of AAV5-mediated gene transfer with etranacogene dezaparvovec, irrespective of pre-existing NAb status.

In terms of long-term safety, 2 recent cases of neoplasm observed among HOPE-B trial participants (1 MDS in 1 NAb+ participant reported herein and 1 glossopharyngeal schwannoma in 1 NAb− participant [28]) were subjected to comprehensive molecular analyses, including assessments of vector integration [28] (Supplementary Methods). The results were consistent with concordant the established preferential hepatic tropism of the AAV5 serotype: there was no evidence of vector DNA in the analyzed non-liver tissues, and no vector integration was detected in tumor samples, thereby excluding vector involvement. These findings align with the low integration frequency characteristic of AAV vectors and are consistent with previously published reports [14]. With more than 20 years of clinical use, AAV-based gene therapy for hemophilia has shown no confirmed cases of AAV-related cancer, despite concerns about potential insertional mutagenesis [24]. To date, multiple independent analyses have failed to implicate AAV vectors in the development of malignancy following systemic administration for hemophilia gene therapy. While long-term follow-up studies continue to further characterize the safety profile and address any latent risks, the accumulated evidence increasingly supports the benign nature of AAV vector integration in this clinical context. As the field approaches more than 2 decades of reassuring clinical experience and robust post-therapy surveillance, it becomes appropriate to focus investigations on confirming the continued absence of vector-related malignancies rather than expecting the emergence of causative evidence. Continued monitoring, including long-term registry follow-up, remains scientifically valuable, especially to strengthen confidence in the long-term safety profile of AAV-based therapies and to support evidence-based risk-benefit assessments and post-marketing strategies.

## 5 Conclusion

This post hoc analysis of the HOPE-B trial provides compelling evidence that etranacogene dezaparvovec is both effective and safe for individuals with hemophilia B and pre-existing AAV5 NAbs, at least up to a titer of 678. Durable transgene expression over 4 years and significant reductions in bleeding rates were seen in NAb+ individuals. Safety remained favorable with no increased risk of liver toxicity or malignancy. This study challenges the traditional exclusion of NAb+patients from gene therapy trials, providing important insights into the feasibility of this specific AAV5-based gene therapy in the presence of pre-existing humoral immunity, and expands eligibility for this therapy to a broader hemophilia B population.
