# Supplementary material for: Etranacogene dezaparvovec in people with hemophilia B with preexisting adeno-associated virus 5 neutralizing antibodies: 4-year subgroup results from the HOPE-B trial
Source: Res Pract Thromb Haemost. 2026 Jan 19;10(1):103360. doi: 10.1016/j.rpth.2026.103360 (PMC12934310; doi:10.1016/j.rpth.2026.103360)
Supplement: Supplementary Data [file mmc1.pdf]

Supplementary materials

Etranacogene dezaparvovec in hemophilia B participants with pre-existing AAV5 neutralizing antibodies: 4-year subgroup results from the HOPE-B trial

Contents

Supplementary Methods: Molecular analysis methodology 2

Supplementary Results: Adjusted ABRs 4

Supplementary Results: Endogenous factor IX (FIX) expression and ALT elevation 6

3. Supplementary text: Narrative for participant with myelodysplastic syndrome (MDS) 7

Analysis of bone marrow aspirates 7

AAV integration studies and molecular characterization 8

Summary with interpretation 9

References 10

Supplementary Methods: Molecular analysis methodology

Molecular analyses were conducted by ProtaGene CGT GmbH (Heidelberg, Germany) independently from the sponsor. DNA isolation of bone marrow tissue and blood, testing for presence of vector in samples, whole genome sequencing (WGS) library preparation, WGS integration site analysis and variant calling were performed as described elsewhere [1].

Briefly, DNA was extracted from tissue and blood using the QIAamp DNA Mini Kit (Qiagen) following the manufacturer's instructions and stored at −20°C for further use. To test for vector DNA presence in samples, a polymerase chain reaction (PCR) with vector-specific primers (hFIXco_FW and hFIXco_RV) was performed on 10 ng DNA per sample, using automated electrophoresis (TapeStation, Agilent Technologies). Vector-containing plasmid was used as a positive control.

WGS library preparation was performed using the TruSeq DNA PCR-free library preparation kit (Illumina). Libraries were sequenced using 150bp paired-end sequencing on a NovaSeq 6000 device (Illumina). WGS sequencing data were analyzed for the detection of integration sites (IS) and to perform somatic variant calling. Briefly, raw sequencing reads were processed for initial alignment with the provided adeno-associated virus (AAV) vector reference sequence (pVD1065) using the BWA-MEM aligner [2]. Subsequently, reads with vector signatures were extracted, and this subset of data was processed for IS analysis with human and vector reference genome (hg38 and vector reference) using the GENE-IS tool suite [3]. Variant calling was done using the Illumina BaseSpace platform (www.illumina.com). DRAGEN Somatic was employed. Standard filters were applied, including 'PASS' to remove likely false positives and 'lowComplexityRegion' to exclude variants in repetitive regions, and finalized using annotation tools, e.g., COSMIC and Ensembl. Somatic variants were called using the DRAGEN Somatic pipeline. Variants passing these criteria were retained for downstream analysis. To filter further, only variants present in established databases (COSMIC, HGNC, Ensembl, and ClinVar) and predicted to have functional impact were considered for interpretation.

In addition, target enrichment sequencing (TES) was used to selectively enrich for AAV vector sequences and determine vector IS. In brief, SureSelect RNA baits (Agilent) were designed covering the entire sequence of the AAV vector using 8x tiling. In addition to the vector-specific baits, baits for two genomic regions of 1 kb length each (chr1: 110,603,487-110,604,486 and chr17: 78,049,323-78,050,322) were designed and included in the bait set. 3 µg of genomic DNA extracted from samples were sheared to a median length of 500 bp using a Covaris M220 instrument and split in 3 technical replicates. Sequencing libraries were prepared using the SureSelect XT HS2 kit. Indexed samples were pooled and

hybridized with SureSelect RNA baits for 90 minutes. Baits and bound target DNA were recovered using streptavidin-coated beads. Unbound DNA was removed, and target DNA was amplified by PCR with Illumina adaptor specific primers. All steps from hybridization to PCR enrichment were repeated to increase capture specificity. The resulting library was sequenced by 250 bp paired-end sequencing on the MiSeq platform (Illumina). Raw sequence data were analyzed using the GENE-IS tool suite [3] to detect and characterize vector IS.

Supplementary Results: Adjusted ABRs

In the full analysis set, adjusted ABRs for all bleeds, joint bleeds, and spontaneous bleeds largely exceeded adjusted ABRs during lead-in for each post-treatment period (**Table S1**). Consequently, non-inferiority of etranacogene dezaparvovec compared to continuous prophylaxis was not met for any of the bleeding categories, with the upper limit of the two-sided 95% Wald confidence interval (CI) exceeding the margin of 1.8. Of note, the higher adjusted ABR during months 7–48 compared to lead-in after treatment with etranacogene dezaparvovec was primarily due to a statistical artifact introduced by the pre-specified data rules of the Statistical Analysis Plan, which dictated that any bleeding events occurring after gene therapy should be imputed to the period of time after gene therapy during which exogenous FIX therapy would not contribute to hemostasis. Time within 5 half-lives of a FIX injection was removed from the time at risk for bleeding per the pre-specified data rules. The participant who received a partial dose of etranacogene dezaparvovec had 7 total (all), 5 joint, and 5 spontaneous bleeding episodes and 0.553 years at-risk time during lead-in compared to 6 total (all), 1 joint, and 1 spontaneous bleeding episodes and 0 years at-risk time (due to contamination) during months 7–48 post-treatment with etranacogene dezaparvovec. To avoid exclusion of the participant's data from the regression analysis, the bleeding event counts and uncontaminated person time from the adjacent 6-month time intervals (after Day 21 through Month 6) was added, leading to 7 total (all), 2 joint, and 2 spontaneous bleeding episodes and 0.055 years at-risk time during months 7–48. The second participant with baseline AAV5 NAb titer 3212 also contributed to the higher adjusted ABR at months 7–48. He had 0 bleeding episodes of all, joint, and spontaneous bleeds and 0.515 years at-risk time during lead-in compared to 5 all, 4 joint, and 4 spontaneous bleeding episodes and 0.003 years at-risk time during months 7–48, resulting in an ABR of >1600. It is understood that an ABR of >1600 is not clinically possible and is a statistical anomaly of the applied methods.

Full analysis set	ABR (95% CI) Lead in n=21	ABR (95% CI) 7-48 months n=21	Two-sided 95% Wald CI n=21
All bleeds	4.84 (3.54, 6.62)	26.03 (5.60, 121.01)	1.14, 25.27
Joint bleeds	3.38 (2.36, 4.83)	14.01 (2.50, 78,39)	0.73, 23.46
Spontaneous bleeds	2.34 (1.36, 4.01)	26.55 (4.29, 164.09)	1.68, 76.82
**Responder analyses set**	**ABR Lead in n=19**	**ABR 7-48 months n=19**	**Two-sided 95% Wald CI n=19**
All bleeds	4.43 (3.17, 6.20)	1.13 (0.60, 2.13	0.14, 0.46
Joint bleeds	3.05 (2.03, 4.56)	0.52 (023, 19)	0.08, 0.34
Spontaneous bleeds	1.96 (1.07, 3.60)	0.41 (0.17, 1.00)	0.10, 0.42

Supplementary Table S1. Adjusted ABRs during months 7–48 post-infusion of etranacogene dezaparvovec compared with continuous prophylaxis during lead-in. ABR, annualized bleeding rate; CI, confidence interval

Full analysis set	ABR (95% CI) Year 1 n=21	ABR (95% CI) Year 2 n=21	ABR (95% CI) Year 3 n=20	ABR (95% CI) Year 4 n=18
All bleeds	2.08 (0.98, 4.38)	1.25 (0.68, 2.27)	1.79 (0.61, 5.23)	0.38 (0.17, 0.89)
Joint bleeds	0.92 (0.42, 1.99)	0.65 (0.28, 1.55)	0.80 (0.25, 2.57)	0.14 (0.04, 0.46)
Spontaneous bleeds	0.56 (0.24, 1.31)	0.49 (0.23, 1.06)	0.98 (0.31, 3.15)	NC*
**Responder analysis set**	**ABR (95% CI) Year 1 n=19**	**ABR (95% CI) Year 2 n=19**	**ABR (95% CI) Year 3 n=18**	**ABR (95% CI) Year 4 n=17**
All bleeds	1.30 (0.63, 2.67)	1.22 (0.66, 2.26)	1.76 (0.60, 5.20)	0.38 (0.16, 0.87)
Joint bleeds	0.61 (1.24, 1.51)	0.64 (0.27, 1.55)	0.78 (0.24, 2.60)	0.14 (0.04, 0.46)
Spontaneous bleeds	0.29 (0.11, 0.76)	0.46 (0.20, 1.04)	0.92 (0.27, 3.12)	NC*

Supplementary Table S2. Adjusted ABRs in years 1–4 post-infusion of etranacogene dezaparvovec. ABR, annualized bleeding rate; CI, confidence interval; NC, non-calculable as there were no spontaneous bleeds during that period of time in any of the participants

Supplementary Results: Endogenous factor IX (FIX) expression and ALT elevation

Supplementary Figure shows endogenous FIX expression over 48 months of follow-up for individual participants with (blue and green colored plots) or without (grey plots) treatment-related alanine aminotransferase (ALT) elevation, as well as the duration of corticosteroid treatment in response to ALT elevation

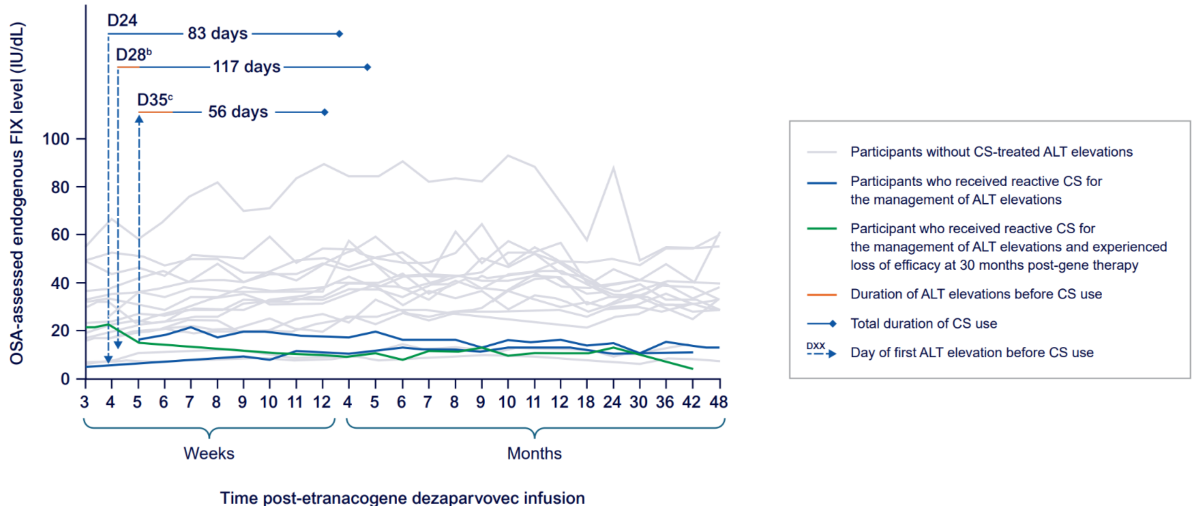

ALT, alanine aminotransferase; CS, corticosteroid; D, day; FIX, factor IX

3. Supplementary text: Narrative for participant with myelodysplastic syndrome (MDS)

The participant was a 59-year-old (at the time of study screening) male with moderately severe hemophilia B (screening FIX activity 2 IU/dL). Past history was significant for an approximately 20-year active hepatitis C that was documented to have resolved five years before enrolment in HOPE-B. The participant had no documented exposure to industrial chemicals, pesticides, smoking, or high dose chemotherapy or radiation. He received the full dose of etranacogene dezaparvovec (study day 0, D0). He presented mild anemia and thrombocytopenia on D1098, judged by the investigator as not related to etranacogene dezaparvovec. The participant was treated with ARANESP 300 µg subcutaneously every two weeks (Q2W) from D1112 to D1554.

Analysis of bone marrow aspirates

A bone marrow (BM) aspiration was performed on D1360. No exogenous FIX replacement therapy was administered prior or after BM aspirations (circulating one-stage FIX activity ~40 IU/dL). Morphology and flow cytometry analysis revealed no significant increase in blast cells, no evidence of monotypic B-cell population, and no aberrant T-cell population. Also, cytogenetic results from MDS fluorescence in situ hybridization (FISH) were within the normal range. However, next generation sequencing (NGS) was performed, and two variants of clinical significance for the development of MDS were detected; Variant allele frequencies (VAF) were 14% for the *STAG2* c.1906 del (p.Y636Tfs*15) and 22% for the *SRSF2* c.284_307 del (p.P95_R102 del), respectively. The molecular findings suggested at minimum a clonal cytopenia of undetermined significance. While significant morphologic dysplasia was not seen (>10% of lineage), close follow-up was recommended.

A follow-up BM aspiration was done approximately 3 months later: cellularity was focally normal, overall noted as hypocellular with no increase in myeloid blast cells. There was no significant dysplasia among erythroid and granulocytic elements. Megakaryocytes exhibited non-specific atypical morphology. NGS confirmed the two variants that had been detected earlier, with increase of VAF to 32% for the STAG2 variant and 28% for the SRSF2 variant. The constellation of the detected variants and atypical megakaryocytes was highly suspicious for low-grade myelodysplastic neoplasm. MDS was reported as an adverse event (AE) on D1534.

AAV integration studies and molecular characterization

To investigate the etiology of MDS focusing on a potential involvement of etranacogene dezaparvovec vector integration into the host genome during oncogenesis, further analysis on samples from the second BM aspirate and whole blood were performed at ProtaGene CGT GmbH (Heidelberg, Germany). First, a semi-quantitative PCR was carried out using vector-specific primers located at the 3’-end of the transgene to test for the presence of vector DNA. No vector signal was detected in this analysis. Despite the absence of vector signal, additional in-depth analysis was done: WGS was applied for identifying somatic variants that had the potential to explain the tumor origin and for the detection of vector IS. In addition, TES was used to assess the presence of integrated vector sequences.

WGS analysis - Somatic variants

The raw reads for BM and blood samples passed quality control. The average base quality was 36/37 and the average coverage was estimated to be 131.6 and 194.66 for blood and BM sample, respectively. The estimated chromosome count was 46.17 and the estimated ploidy was 1.95 for both samples. The purity of the tumor sample could not be reliably estimated, as the absence of a matched normal sample makes it difficult to distinguish true variants from background. No copy number variants (CNVs) were detected. Somatic structural variant analysis (SSV) retained 26 and 28 variants in the blood and in the BM sample, respectively, but none of them were reported as drivers for MDS disease [4]. In somatic number variants (SNV) analysis, more than 270,000 variants were identified in the blood and BM samples after applying the filters. To account for the unavailability of match normal sample, only variants with VAF <0.5 were retained, as higher frequencies are more likely to represent germline events. Focusing on missense and frameshift variants, the same set of variants was identified in both blood and BM, except for *TP53*, which showed a VAF <0.5% in blood. ClinVar annotation was used to exclude known germline variants. After applying this filtering, the same two variants as detected by NGS analysis were found in both blood and BM samples, with VAF at 43% for the *STAG2* variant, and 38% for the *SRSF2* variant. One variant on chromosome X (position 124,063,930) was identified as a CT dinucleotide deletion within *STAG2*. This frameshift mutation introduces a premature stop codon and is classified as high impact, likely leading to loss of function. *STAG2* (Xq25) is the most frequently mutated cohesin complex gene in myeloid neoplasms, particularly MDS and acute myeloid leukemia (AML), and somatic *STAG2* variants are considered ‘secondary-type’ mutations [5, 6]. The variant is also catalogued in COSMIC (COSV54350437), linked to Ewing sarcoma, and occurs in an enhancer region that may influence transcriptional regulation. The presence of this variant with VAF ~43% is inconsistent with a typical germline

X-linked variant, which in males would appear hemizygous at ~100%. This strongly suggests that it is a true somatic variant.

The second variant was identified on chromosome 17 at position 76,736,853. It affects the *SRSF2* gene, leading to an inframe deletion of amino acids Pro95_Arg102, and is classified as of moderate impact variant due to its potential disruption of protein function. *SRSF2* is known to play a critical role in RNA splicing and gene expression regulation (Liang et al. 2018). Mutations in *SRSF2* have been frequently associated with hematologic malignancies, including MDS and AML. The inframe deletion observed likely affects the splicing function of *SRSF2*, contributing to the pathogenesis of these cancers. This variant was detected with a VAF ~36%, a frequency inconsistent with a typical germline heterozygous pattern (expected ~50%).

IS analyses by WGS and TES

WGS data from bone marrow and peripheral blood were processed for IS retrieval with human reference genome (hg38) and the provided pVD1065 vector sequence. However, no IS were detected in either sample. As WGS – although with high coverage – can only detect IS in the low percentage range, the same samples were analyzed by TES, a targeted approach to identify vector sequences integrated into the host genome. TES of genomic DNA extracted from bone marrow detected no insertion sites. Analysis of genomic DNA extracted from peripheral blood samples detected a single sequencing read that classified as one IS in only one out of three replicates. The nearest gene detected was *FAM78A* (chr9:131278140), not associated with MDS or other hematopoietic disorders. Thus, TES analysis showed no dominant IS present in the analyzed samples.

Summary with interpretation

Taken together, the molecular analysis detected two clinically relevant somatic variants: a premature stop codon in *STAG2* (VAF ~43%) and an inframe deletion in *SRSF2* (VAF ~36%). Both variants are established contributors to MDS [5]. In contrast, no vector IS could be detected in bone marrow, neither by WGS nor TES, and only a single inconclusive IS was detected in blood, implying that the tumor origin was unrelated to etranacogene dezaparvovec therapy.

References

1. Raheja P, O'Connell N, Kampmann P, Lemons R, Wang F, Gill S, et al. PLACEHOLDER: Etranacogene dezaparvovec in participants with hemophilia B and without adeno-associated virus serotype 5 neutralizing antibodies: A 4-year subgroup analysis (HOPE-B) rpth. 2025.
2. Li Y, Chen L, Shao D, Zhang B, Xie S, Zheng X, et al. Hereditary intraspinal schwannomatosis with SMARCB1 gene mutation: A case report. J Clin Lab Anal. 2022;36(6):e24448.
3. Afzal S, Wilkening S, von Kalle C, Schmidt M, Fronza R. GENE-IS: Time-Efficient and Accurate Analysis of Viral Integration Events in Large-Scale Gene Therapy Data. Mol Ther Nucleic Acids. 2017;6:133-9.
4. Ogawa S. Genetics of MDS. Blood. 2019;133(10):1049-59.
5. Haferlach C, Stengel A, Meggendorfer M, Kern W, Haferlach T. Characterization of MDS Harboring TET2 Mutations and/or TET2 Deletions. Blood. 2016;128(22):4288-.
6. Katamesh B, Nanaa A, He R, Viswanatha DS, Nguyen PL, Greipp P, et al. Clinical and Genetic Characteristics of STAG2 Mutations in Myeloid Neoplasms. Blood. 2022;140(Supplement 1):12353-4.
